# Suicide: can we identify and manage those at risk more effectively?

**DOI:** 10.1192/bjo.2021.906

**Published:** 2021-06-18

**Authors:** Emily Roberts, Anne-Marie Grew, T Everett Julyan

**Affiliations:** 1 University of Glasgow; 2 NHS Ayrshire & Arran

## Abstract

**Aims:**

This study aimed to conduct longitudinal analysis of suicide reviews for mental health service users in Ayrshire to improve local practice and outcomes. Traditional risk factors – middle-age, male and alcohol misuse – were hypothesised to convey greater risk of completing suicide.

**Background:**

Suicide is an important public health issue in Scotland, with potentially devastating impacts. Practice and policy may lag behind emerging evidence. Mental health problems are associated with an increased suicide risk, and care provided to those who take their own lives is reviewed to identify recommendations and learning points to improve practice and outcomes. However, these reviews and their conclusions are often considered individually, when studying them collectively over time it is necessary to characterise common themes and highlight factors that could be addressed to reduce suicide. Moreover, national averages can obscure local patterns.

**Method:**

Access to reviews of suicides for mental health service users in Ayrshire was granted by the Adverse Event Review Group. Relevant data were extracted for the 35 General Adult service users completing suicide between 2013 and 2015, including details of the act, demographics and clinical factors, and analysed for trends. Those with and without emotional instability as a primary diagnosis or significant problem were dichotomised to facilitate identification of statistically significant factors specific to these symptoms.

**Result:**

There were 35 completed suicides including three inpatients. Suicide was most common in the 25-29 and 45-54 age ranges, and over 68.6% were male. Hanging accounted for 60.0% of deaths, and self-poisoning for 8.6%. Up to 62.9% of patients did not appear to have ongoing scheduled appointments on a regular basis. Diagnoses were difficult to identify – 48.6% had no clear primary diagnosis specified in the reviews, and features of depressive, anxiety, psychotic, substance misuse and personality disorders frequently overlapped and co-occurred. 22.9% had problems with emotional instability; their median age was 14 years younger, and 87.5% were female.

**Conclusion:**

Small sample size precluded detailed analysis. The traditional risk profile remains relevant. However, almost 25% of those completing suicide were younger females with emotional instability, despite frequent contact with services. Given the challenges in predicting suicide, we should continue to consider how best to prevent this tragic outcome in all service users, especially in younger females with emotional instability; middle-aged males who misuse alcohol, and those with ill-defined diffuse psychological difficulties who do not fit into discrete categories or are reviewed infrequently.

